# Neither sulfoxaflor, *Crithidia bombi*, nor their combination impact bumble bee colony development or field bean pollination

**DOI:** 10.1038/s41598-023-43215-6

**Published:** 2023-09-30

**Authors:** Edward A. Straw, Elena Cini, Harriet Gold, Alberto Linguadoca, Chloe Mayne, Joris Rockx, Mark J. F. Brown, Michael P. D. Garratt, Simon G. Potts, Deepa Senapathi

**Affiliations:** 1 Department of Botany, Trinity College Dublin, Dublin, D02 PN40 Ireland; 2 Department of Biological Sciences, Centre for Ecology, Evolution and Behaviour, School of Life Sciences and the Environment, Royal Holloway University of London, Egham, Surrey TW20 0EX UK; 3 Centre for Agri-Environmental Research, School of Agriculture, Policy and Development, University of Reading, Reading, RG6 6AR UK; 4 The School of Archaeology, Geography and Environmental Sciences, University of Reading, Reading, RG6 6AB UK; 5 Pesticides Peer Review Unit, European Food Safety Authority (EFSA), Via Carlo Magno 1A, 43126 Parma, Italy; 6 School of Biological Sciences, University of Reading, Reading, RG6 6AS UK

**Keywords:** Agroecology, Ecosystem services, Environmental impact

## Abstract

Many pollinators, including bumble bees, are in decline. Such declines are known to be driven by a number of interacting factors. Decreases in bee populations may also negatively impact the key ecosystem service, pollination, that they provide. Pesticides and parasites are often cited as two of the drivers of bee declines, particularly as they have previously been found to interact with one another to the detriment of bee health. Here we test the effects of an insecticide, sulfoxaflor, and a highly prevalent bumble bee parasite, *Crithidia bombi*, on the bumble bee *Bombus terrestris*. After exposing colonies to realistic doses of either sulfoxaflor and/or *Crithidia bombi* in a fully crossed experiment, colonies were allowed to forage on field beans in outdoor exclusion cages. Foraging performance was monitored, and the impacts on fruit set were recorded. We found no effect of either stressor, or their interaction, on the pollination services they provide to field beans, either at an individual level or a whole colony level. Further, there was no impact of any treatment, in any metric, on colony development. Our results contrast with prior findings that similar insecticides (neonicotinoids) impact pollination services, and that sulfoxaflor impacts colony development, potentially suggesting that sulfoxaflor is a less harmful compound to bee health than neonicotinoids insecticides.

## Introduction

Pollinators, principally bees, are responsible for $235–577 billion a year in crop production^1^, including many nutritionally rich crops like fruits, vegetables, and nuts^2,3^. However, pollinators, and bees specifically, are thought to be suffering population declines globally^4–6^. The threats to bees are multifactorial and interactive, with habitat destruction/fragmentation^7–9^, intensive agriculture^10^, and climate change^11,12^, all cited as drivers of decline. A key potential driver of bee declines linked to agriculture is the use of pesticides, which are important tools for farmers in protecting crop yields and farmer profits^13^. While in some instances, pesticides can be more environmentally friendly than non-chemical interventions^14^, and despite the economic benefits of pesticides, they have been implicated in driving bee declines^15–17^.

Recent focus on the effects of pesticides on bees has begun to shift towards how multiple stressors affect bee health^18–21^. This is because, in the wild, bees face a diverse array of stressors, including nutritional deprivation^22^, predation^23^, pesticides^24^, and parasites^25^, and it is not uncommon for bees to face more than one of these stressors at any one time^26,27^. Research quantifying the impacts of combined parasite-pesticide exposure on bee health has found synergies, whereby combined exposure exceeds the cost of each stressor individually^28^. However, not all combinations act synergistically, or cause any additional harm^19,20^.

Bumble bees, *Bombus spp.*, are some of the most important bee species contributing to crop pollination in Europe and North America^29^. Managed colonies are used for commercial pollination of fruits, particularly in greenhouses^30^, and wild bumble bees also contribute significantly to field crop and orchard pollination^31,32^. Bumble bees have also been shown to contribute to the yield of important crops such as oilseed rape and field beans^33,34^. Consequently, understanding and ultimately mitigating the impact of multiple interacting stressors on bumble bee health is key to maintaining their contribution to pollination in agri-ecosystems.

Previous studies have shown that the highly prevalent gut parasite *Crithidia bombi*, despite being relatively benign in favourable circumstances, has significant impacts on bumble bee health, such as lower colony reproduction and fitness^35–37^, impaired cognitive abilities^38^, and foraging behaviour^38–41^. It is unknown if other stressors, like agrochemical exposure, would create conditions in which *C. bombi* would become more detrimental to colony and worker health. Similarly, studies of agrochemicals that act as selective agonists of nicotinic acetyl choline receptors in insects (*e.g.*, neonicotinoids and sulfoximines) have shown that they have detrimental effects on pollination services^42^, as well as effects at the individual and colony level, including for foraging behaviour^19,43^, and memory and learning abilities^44–46^. As such, investigating the impact of other classes of insecticides on bee health is key to finding safer and effective alternatives for crop pest management^47,48^.

Over the past few years, research has started to better explore the insecticide sulfoxaflor and its effect on bees. Sulfoxaflor is the first marketed insecticide belonging to the sulfoximines group, and despite having been linked to lower worker production and reproductive success of bumble bee colonies (similar to neonicotinoids^47^), and to a lower egg production^49^, no effect of chronic exposure to this substance has been found on bumble bee foraging performance^47^, and no impact of acute exposure on bee learning and behaviour has been observed^50^. This is contrary to neonicotinoids at comparable dosages^42,45^.

Although existing research has investigated the interaction effect of *C. bombi* with some common insecticides^51–53^, no study has yet analysed its effect in combination with sulfoxaflor. With this study, we aim to build on earlier single-stressor experiments to ask how parasites and pathogens impact pollination services and colony development in this important pollinator, addressing research gaps on potential interactive effects of sulfoxaflor with *Crithidia bombi* on bee health and crop pollination.

## Methods

An overlapping experimental block design was employed for this study, with the same set of procedures taking place at the same time intervals for each experimental block. There were nine experimental blocks staggered over six weeks from April 2021 through to June 2021. Each block included eight colonies except the first experimental block, which comprised four colonies. Young *Bombus terrestris audax* colonies were ordered in batches from Agralan (Swindon, UK), and each was contained in a cardboard box (30 × 20 × 24 cm) equipped with an extendable ventilation system and a feeding system comprising a bottle of 2.1 kg of glucose. They were maintained on honey bee-collected pollen and 50% (w/v) sucrose in darkness at the Royal Holloway University of London.

### Screening

On day 1, under red light and using forceps, 20 bees were removed from each colony, induced to defecate, and returned to the colony. Their pooled faeces was screened for parasites^27^ to ensure the colonies were uninfected by microparasites.

### Colony standardisation

On day 2, all the bees were removed from the colony, except for the queen. Afterwards, 20 bees were returned to the colony, chosen haphazardly. The resulting colony was then weighed and returned to its feeder. Colonies were allocated to treatments using a weight rank allocation, rotated between experimental blocks.

### *Crithidia bombi* inoculation

On day 3, between 30 to 40 workers were removed from two colonies deliberately infected with *Crithidia bombi* (these functioned as parasite reserve colonies and are distinct from the experimental colonies), originally sourced from infected wild queens caught in Windsor Great Park in 2021. The faeces from these bees was pooled and purified^54^. The inoculum concentration was quantified using a Neubauer haemocytometer and diluted in 1 mL water to a dose of 525,000 cells, equivalent to 25,000 cells per bee (20 workers and the queen), a dose which induces a realistic infection. Four mL of 40% w/w sucrose was added and the inoculum was vortexed. The inoculum was pipetted into a 35 mm petri dish and presented to the colonies for 24 h, or 48 h if not consumed within the first 24 h. Control bees were exposed to 4 ml 40% w/w sucrose and 1 mL water. Once the inoculum was visually verified as consumed, bees were returned to their standard feeder. Colonies were left to develop for a week, allowing the infection to take hold.

### Validation of infection

On day 10, colonies were screened for the presence of *C. bombi* to ensure successful inoculation, and that the control colonies were not accidentally infected. To achieve this, 30 workers (or all workers if < 30 were present) were removed from a colony and induced to defecate. Their faeces was screened for *C. bombi* presence/absence. An arbitrary 25% prevalence rate was chosen as our threshold for a successful infection (50% mean average) with no colonies discarded. If a Control or Sulfoxaflor-only colony had even a single instance of *C. bombi*, the whole colony was discarded (*n* = 2). The following day (day 11), colonies were driven from Royal Holloway to the University of Reading, with a travel time of around an hour, during which their sucrose reservoirs were closed. On day 12, colonies were rested for a day to settle in a well-ventilated room with controlled temperature (24–26 °C) and humidity (50 ± 20%).

### Pesticide exposure

On day 13, the chronic sulfoxaflor exposure scenario was modelled after residue data from a study submitted as part of the assessment of the confirmatory data for sulfoxaflor in the European Union (EU)^55^. The residue study (trial S16-00602) applied sulfoxaflor as 24 g active ingredient/ha as GF-2626 (a sulfoxaflor formulation containing 125 g/L active ingredient) to strawberry crops in semi-field conditions in Southern Germany.

The concentration of sulfoxaflor was measured in bumble bee collected nectar 1, 3, 5, and 7 days after the application. The residue study is a worst-case exposure scenario because mitigation measures were not followed, meaning the crop was sprayed while still in bud, which is not legal in the EU for sulfoxaflor^56^. As such, the exposure we model here represents among the worst-case realistic exposures to sulfoxaflor possible from a single spray without direct exposure to the sprayed liquid. We excluded day 0 of exposure and modelled the following four days, as the residues quickly declined after this to non-quantifiable limits.

The sulfoxaflor exposure used for our experiment used a stepwise degradation profile, as in the ‘best-case’ scenario used in Linguadoca et al.^57^. Here, the pesticide solutions are swapped daily to a new, lower concentration, which mimics the natural degradation of the substance in the field. The concentrations used each day were 161 µg/kg, 47 µg/kg, 14 µg/kg, and 4 µg/kg. Bees were fed either the spiked or unspiked sucrose solution (30% sugar weight/volume), ad libitum, through a gravity feeder.

### Semi-field experiment

Using these colonies, a semi-field experiment was conducted at the University of Reading between May and June 2021 assessing (i) individual and (ii) colony behaviour of bumble bees foraging on field beans in outdoor flight cages, and (iii) the pollination function in terms of field bean yield. Spring field beans (*Vicia faba*) of the ‘Fuego’ variety supplied by commercial seed company Limagrain UK were used for the study. Beans were sown in 3L pots containing ‘John Innes n°2’ compost and thinned to one plant per pot when they reached an adequate size. *Bombus terrestris* are among the most common visitors of *Vicia faba* in the fields^31,58^, therefore investigating changes in their behaviour due to a range of interactive threats is of considerable importance. Moreover, field beans have been shown to benefit from bee pollination, with a higher pod set^31^, pod number^59^, and plant weight^58^, and reduced yield losses under heat stress^34^.

All relevant institutional, national, and international guidelines and legislation was adhered to in the purchase and rearing of *V. faba* plants. No wild plants were used, nor any endangered species. Eight outdoor flight cages (each 4.2 × 2.1 × 4.2 m) were randomly assigned one bumble bee colony for each experimental block where they were left for the entire four-day behavioural observation period. A cage rotation system was in place so that by the end of the trial every treatment had been allocated to all cages at least once.

On day 13, colony boxes were covered with thick layers of cotton wool to help protect them from the cold. They were then placed in a flight cage with designated field bean plants. Behavioural observations were carried out over days 13–16. While in cages, colonies were supplied with fresh ad libitum 30% w/w sucrose solutions every 24 h. The solutions for the ‘Control’ and *‘C. bombi’* groups contained sucrose and water only, while the solutions for the ‘Sulfoxaflor’ and ‘Sulfoxaflor + *C. bombi’* groups also contained the pesticide. Solutions were provided each morning through gravity feeders attached at the base of the box.

### Behavioural observations

**Figure 1:**
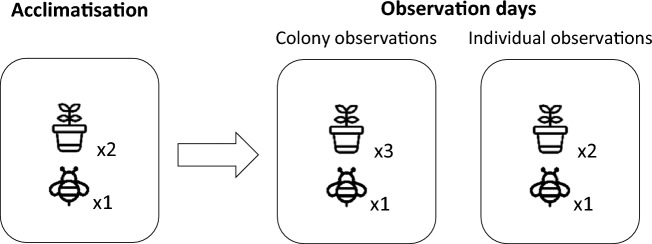
Summary of behavioural observation procedures showing the number of plants in each cage, always containing one colony.

Observations of bee behaviour were based on the work of Stanley et al.^42^. One colony and five field bean plants were used in each of the eight flight cages per study block for four days (Fig. 1). Specifically, on day 13, bees were left to acclimatise to cages for six hours with two field bean plants, after which colonies were closed. On observation day 14, 15, and 16, five field bean plants were moved into the flight cages each day, with three being used for colony observations and two for individual observations. All flowers on each field bean plant were counted to enable calculation of colony visitation rates (average flowers per plant = 182.5).

### Colony observations

Colony activity was measured by filming (using a digital camera placed on a tripod) and later scoring the number of bees leaving and returning to the colony using the event-logging software ‘BORIS’^60^. The observer opened the colony entrance and allowed 10 min of acclimatisation starting from the moment the first bee left the colony, then turned on the camera to record the entrance of the colony. The number of visits made by bees to three field bean plants was also recorded for five minutes per plant for a total of 15 min, before returning the bees to the colony and closing the entrance. The same three plants were exposed to the same colony throughout the three days of observations, and all plants in different cages were exposed to colonies for the same amount of time (25 min/day).

### Individual observations

Two field bean plants were assigned to one colony for individual observations throughout the three-day observation period. The observer allowed one bee out of the colony at a time and recorded its behaviour for a maximum of 15 min starting from the moment it left the colony, aiming for three bees per colony. Recorded behaviours included latency (time taken to visit the first flower), overall duration of foraging trip, time spent on each flower, time between one flower visit and the next, and if pollen was collected or not. Again, observations of individual bees were carried out using ‘BORIS’^60^.

### Pollination service

The three plants used for colony-level observations were also employed as phytometer plants, defined here as plants grown under the same conditions and used to assess the level of pollination delivered by each colony. One stem of each phytometer plant was marked with cable ties above and below the two floral nodes with the freshest and most receptive flowers being visited by bumble bees during the colony observations. After performing colony observations, the plants were transferred to an insect-free flight cage where they continued to grow and ripen for 2 months. At harvest, the number of pods per node between cable ties and node location was recorded, and pods were then dried in the oven for 48 h at 80 °C, after which pod weight, number of beans per pod, and weight of individual beans was recorded.

### Colony development

On day 16, following the final day of observations, colonies were returned to a temperature-controlled room, and left on ad libitum unspiked sucrose (50% w/w) and pollen (Agralan), topped up weekly. Colonies were kept for a total of six weeks after the pesticide exposure, and then frozen at – 20 °C (day 58). Colonies were returned to Royal Holloway and dissected in three batches. The total number of workers, males, queens, gynes, larvae, and pupae were recorded per colony, alongside the weight of the workers, males, queens, gynes and larvae.

### Statistical analysis

#### Blinding

The entire University of Reading team was blinded to the treatments until after the experiment had concluded. To facilitate this, colonies and pesticide solutions were labelled using alphabetical codes corresponding to the treatment.

#### Semi-field experiment

A total of 88 colony observations, 149 individuals, and 106 plants were successfully analysed (Table 1). Due to heavy rain and low temperatures, four experimental blocks had to be excluded from all analyses. Further, as the poor weather did not allow us to perform all individual observations in block one, this additional block was discarded from the individual observation analysis. Moreover, 21 files with data on number of bees leaving and re-entering colonies were lost, and thus could not be included in the analysis. Such files were randomly distributed across plants and treatments. Finally, one of the phytometer plants died and was thus discarded.

**Table 1:** Details on sample sizes of colony observations (visitation rates and bees leaving/returning to the colony), individuals, and employed plants across colonies included in the analysis.

Treatment	N colony observations (visitation rate)	N colony observations (bees leaving/returning to colony)	N individuals	N plants
Control	23	19	42	26
*C. bombi*	23	17	43	26
Sulfoxaflor	22	16	31	27
*S*ulfoxaflor + *C. bombi*	20	15	33	27
Total	88	67	149	106

Colony and individual observation data came from 34 and 27 colonies respectively.

Data were checked for correlations using the Pearson Product-Moment test, and linear and generalised linear mixed-effect models were built in Genstat 21st Edition^61^ to assess the impact of treatments on bee behaviour and plant yield. The models with the lowest AICc value and ΔAICc ≤ 2 was selected as the model with the most explanatory power^62,63^. Fisher’s protected LSD post-hoc tests were planned in case observation day or treatment would have been significant. Parameter estimates and standard errors are presented in the supplementary materials for the semi-field experiment work.

##### Individual and colony observations

Response variables for individual observations included duration of foraging trip, foraging rate, and pollen collection, with visitation rate used as a dependent variable for colony-level assessments (see Supplementary Materials for further metrics). The model (Metric ~ Treatment * Observation Day + (1|block and colony ID + observer)) was used. Treatment, observation day, and interaction between the two were included as fixed terms, while ‘experimental block and colony ID’ and ‘observer’ were included as random factors. Data were analysed using Linear Mixed Models (LMMs).

##### Pollination services

Response variables for pollination services included average number and weight of pods between cable ties, and were analysed using LMMs. The model (Metric ~ Treatment + First node location + (1|block and colony ID/plant ID)) was used. Treatment and location of first node were included as fixed terms, while ‘plant’ nested within ‘experimental block and colony ID’ were used as random factors. ‘First node location’ was treated as a categorical variable including early (1 to 5), middle (6 to 10), and late flowering nodes (11 to 16).

### Colony development

Statistical analyses were carried out in ‘R’ programming software version 3.6.2^64^. Worker weight, larval weight, and number of pupae were analysed using LMMs, utilising the package ‘lme4’^65^. Model assumptions were tested and met. The model (Metric ~ Treatment + Mass of bees upon delivery + (1|block)) was used. Mass of bees upon delivery was included as a fixed factor to account for the variation in the size of the colonies, which reflected the eggs, larvae, and pupae they had after standardisation. Larval weight and drone weight were analysed using a Kruskal–Wallis model, because the data were non-normal. The model (Metric ~ Treatment) was used, with a Benjamini–Hochberg *p*-value correction applied to account for multiple testing. Post-hoc testing is presented in the supplementary materials.

## Results

### Colony behaviour

**Figure 2:**
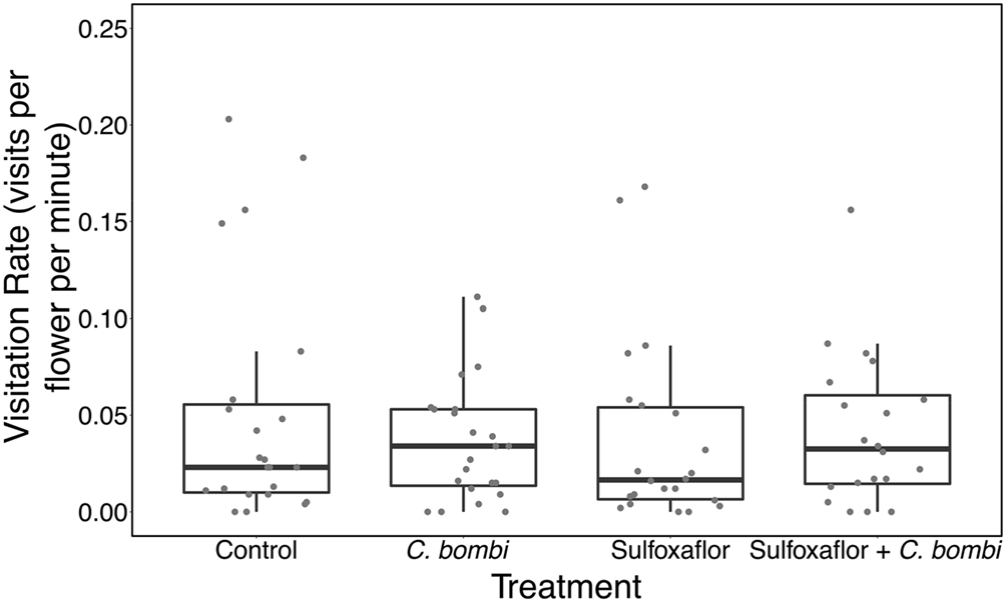
A boxplot with horizontally jittered datapoints showing mean flower visitation rate (visits per flower per minute) by treatment. Bold line is the median, the box is bounded by the first and third quartile of the data, and the whiskers are the 95% confidence intervals.

There was no treatment effect on visitation rate of colonies to field beans (Fig. 2), but a significant effect of observation day was found (Table 2), with the lowest visitation rate on day 14 (see Supplementary Materials for further analyses).

**Table 2:** Analysis of colony behaviour metrics: visitation rate.

Statistical test	Model	Significant effect of any treatment	F, degrees of freedom	p-value	R^2^
LMM	Visitation rate ~ observation day + (1|block and colony ID + observer)	No (not included in final model)	F = 9.43 DF = 2,53.9	< 0.001	18.18

### Individual behaviour

**Figure 3:**
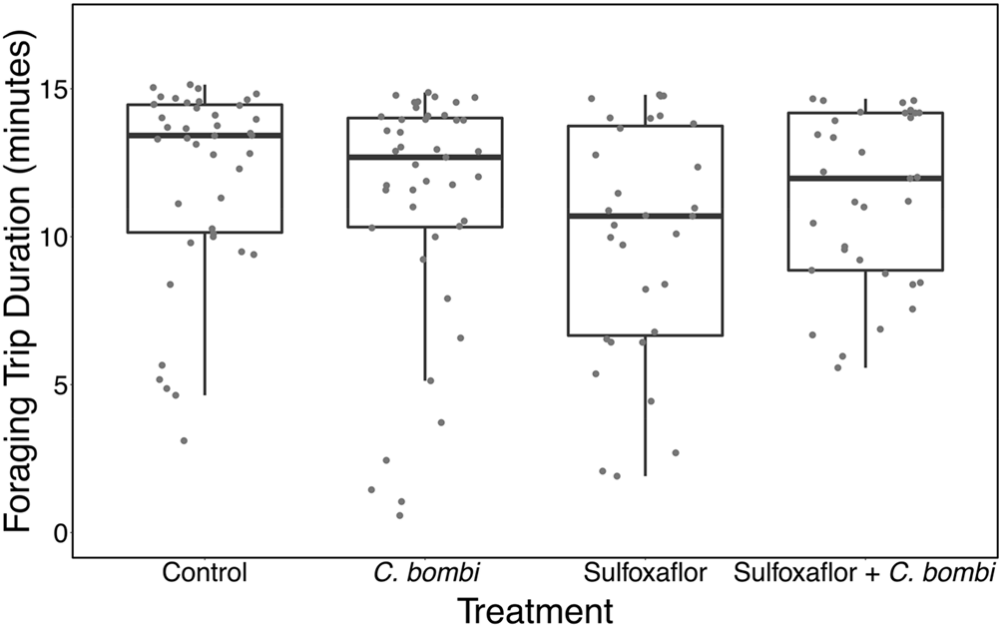
A boxplot with horizontally jittered datapoints showing the mean duration of a foraging trip (in minutes) per bee, by treatment. Bold line is the median, the box is bounded by the first and third quartile of the data, and the whiskers are the 95% confidence intervals.

No significant effect of treatment, observation day, or interaction between the two was found on any metrics related to individual behaviour during foraging (Table 3 and Fig. 3, see Supplementary Materials for further analyses).

**Table 3:** Analysis of individual behaviour metrics: foraging trip, foraging rate, and pollen collection.

Statistical test	Model	Significant effect of any treatment	F, degrees of freedom	p-value	R^2^
LMM	Duration of foraging trip ~ treatment * observation day + (1|block and colony ID + observer)	No	Observation day F = 0.56, DF = 2,124.2 Treatment F = 0.81 DF = 3,20.2 Interaction F = 1.66 DF = 6,109.9	Observation day P = 0.575 Treatment P = 0.504 Interaction P = 0.138	8.69
LMM	Foraging rate ~ treatment * observation day (1|block and colony ID + observer)	No	Observation day F = 0.49 DF = 2,129.6 Treatment F = 0.51 DF = 3,21.5 Interaction F = 1.74 DF = 6,102.7	Observation day P = 0.614 Treatment P = 0.677 Interaction P = 0.119	8.99
GLMM	Pollen collection ~ treatment + observation day + interaction + (1|block and colony ID + observer)	No	Observation day F = 0.73 DF = 2,124.9 Treatment F = 0.89 DF = 3,19.7 Interaction F = 2.35 DF = 6,35.6	Observation day P = 0.484 Treatment P = 0.463 Interaction P = 0.051	19.95

### Pollination services

**Figure 4:**
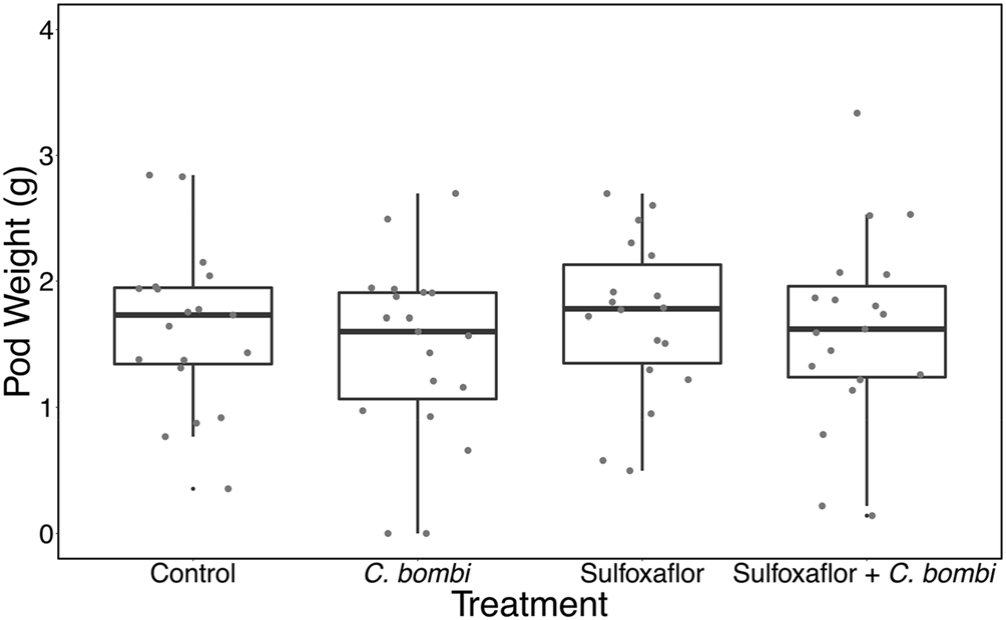
A boxplot with horizontally jittered datapoints showing the mean pod weight of field beans (in grams) by treatment. Bold line is the median, the box is bounded by the first and third quartile of the data, and the whiskers are the 95% confidence intervals.

No significant effect of treatment or location of first node was shown on field bean pollination variables (Table 4 and Fig. 4, see Supplementary Materials for further analyses).

**Table 4:** Analysis of pollination metrics: number of pods and pod weight.

Statistical test	Model	Significant effect of any treatment	F, degrees of freedom	P-value	R^2^
LMM	Average number of pods ~ treatment + first node location + (1|block and colony ID/plant ID)	No	Treatment F = 1.63 DF = 3,31.6 First node location F = 0.19 DF = 2,92.4	Treatment P = 0.203 First node location P = 0.831	4.99
LMM	Average pod weight ~ treatment + first node location + (1|block and colony ID/plant ID)	No	Treatment F = 0.33 DF = 3,24.9 First node location F = 0.51 DF = 2,67.7	Treatment P = 0.803 First node location P = 0.604	2.83

### Colony development

**Figure 5:**
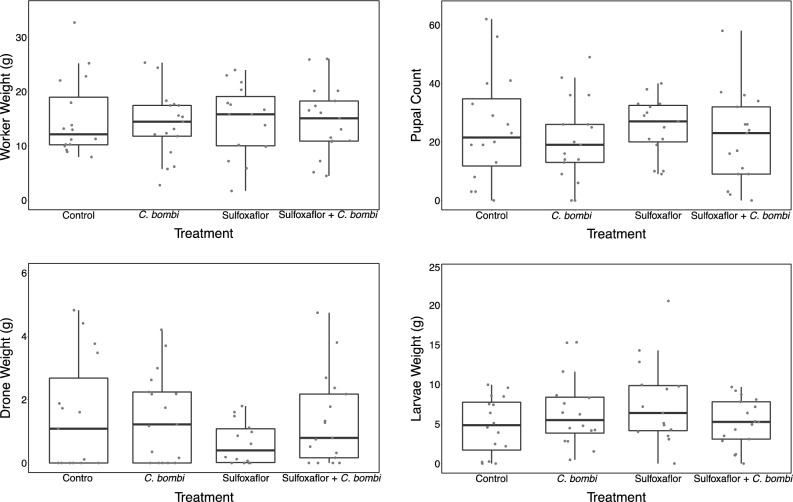
A panel of boxplots with horizontally jittered datapoints. Top left: Cumulative weight of workers in a colony (in grams); Top right: number of pupae in a colony, Bottom left: cumulative weight of males (drones) in a colony (in grams); Bottom right: cumulative weight of larvae in a colony (in grams). Each boxplot shows the metric by treatment. Bold line is the median, the box is bounded by the first and third quartile of the data, and the whiskers are the 95% confidence intervals.

No significant effect of treatment was found for any metric (weight of workers, weight of larvae, weight of drone or number of pupae) (Table 5 and Fig. 5).

**Table 5:** Analysis of colony development metrics: weight of workers, weight of larvae, weight of drones and number of pupae.

Statistical test	Model	Significant effect of any treatment	Parameter estimate (PE) + confidence intervals (CI)	Chi-squared, degrees of freedom, P-value
LMM	Weight of workers ~ treatment + colony weight + (1|block)	No	*Crithidia* only PE = − 0.947 (CI = − 3.832 to 1.976) Sulfoxaflor only PE = − 0.675 (CI = − 3.679 to 2.306) Sulfoxaflor + *Crithidia* PE = − 0.486 (CI = − 3.357 to 2.410)	
Kruskal–Wallis	Weight of larvae	No		X^2^ = 2.461 DF = 3 P = 0.483
Kruskal–Wallis	Weight of drones	No		X^2^ = 2.139 DF = 3 P = 0.544
LMM	Number of pupae ~ treatment + colony weight + (1|block)	No	*Crithidia* only PE = − 1.313 (CI = − 4.883 to 2.256) Sulfoxaflor only PE = 0.672 (CI = − 3.017 to 4.363) Sulfoxaflor + *Crithidia* PE = − 1.065 (CI = − 4.624 to 2.493)

## Discussion

Using a well replicated methodology and tracking a number of different outcomes, we find no evidence for negative effects of sulfoxaflor, *Crithidia bombi,* or their interaction on our measures of bumble bee behaviour, bean pollination, or colony growth and rearing of sexuals. Our results indicate that sulfoxaflor may not be as harmful to bumble bee pollination provisioning as the neonicotinoids thiamethoxam and imidacloprid^42,66^, and that the conditions under which sulfoxaflor impacts reproduction may be narrower than previously expected^47^. Further, the presence of a parasitic stressor does not necessarily lead to the emergence of agrochemical impacts.

Our semi-field experiment found no significant impact of sulfoxaflor at a field-realistic level of exposure on either colony or individual behaviour of bumble bees. Exposure to neonicotinoids, a related group of systemic agrochemicals that act as selective agonists of nicotinic acetyl choline receptors, has been linked to a reduction in bee visitation rate and pollen collection^42,67–69^, which translates into less efficient flower visiting behaviour resulting in a lower production of seeds^42^. In contrast, our results are in line with previous studies, where sulfoxaflor did not impair bee behaviour^50,70^ or pollination by honeybees^71^, although impacts on bumble bees have been found^72^. This difference in behavioural impacts between neonicotinoids and sulfoxaflor may explain the lack of an effect of sulfoxaflor on the yield of field bean plants in our experiment. Indeed, directly comparable testing of a neonicotinoid and sulfoxaflor found sulfoxaflor to have weaker impacts on locomotive behaviour^66^. Alternatively, small differences in our experimental paradigm to that of Stanley et al.^42^, with different exposure regimes and subsequent behavioural monitoring, may explain the difference in our results. However, the complete lack of any trends in colony behaviour, individual behaviour, or pollination services with respect to experimental treatments suggests that differential agrochemical impacts is the most likely explanation.

We also found no impact of *C. bombi* on bee behaviour and pollination, contrary to previous studies where an effect on visitation rate and time spent on artificial flowers was found, resulting in an impairment of foraging abilities^40,41^. Consequently, under our experimental conditions, these laboratory effects do not appear to extrapolate to real-world bee-flower interactions. Finally, the absence of a sulfoxaflor-*C*. *bombi* interaction effect on foraging behaviour or pollination, in this semi-field realistic experiment, mirrors the results of a recent laboratory study^70^, which found no interactive effect of sulfoxaflor and *C. bombi* on bumble bee olfactory learning. Together, this suggests that sulfoxaflor, either on its own or in combination with a highly prevalent parasite, may be a less harmful alternative to neonicotinoids.

In addition to measuring behaviour and pollination services, we also found that neither sulfoxaflor, *C. bombi*, nor their interaction, impacted *B. terrestris* colony development. No effects, or unsupported data trends, were seen regarding the production of any caste or development stage, giving confidence in the result. Most previous studies investigating the impact of sulfoxaflor or *C. bombi* on colony development exposed queens before hibernation or exposed very young colonies^35,36,47^. One study with a similar experimental design is Fauser-Misslin et al.^52^, where exposure to both *C. bombi* and the agrochemical occur at a later stage in the colony life cycle. In contrast to our results, they found significant impacts of the neonicotinoids thiamethoxam and clothianidin on colony growth. They also found an interactive effect of these agrochemicals and *C. bombi* on mother queen survival. Together, this suggests that exposure to sulfoxaflor later in the season is less likely to reduce bumble bee colony health, and that sulfoxaflor is also less toxic to bumble bees than either thiamethoxam or clothianidin. However, we note that our experimental colonies had access to ad libitum food and were largely kept in controlled environmental conditions, in contrast to Siviter et al.^47^, where colonies were exposed to naturally fluctuating climatic conditions and had to forage for food. Consequently, it is possible that the semi-field experimental setup could have contributed to decreasing the impact of sulfoxaflor on the colony compared to an uncontrolled environment, such as an open crop field. Again, however, in Fauser-Misslin et al.^52^ neonicotinoids still had a negative impact despite such buffering, suggesting that neonicotinoids are indeed more toxic to bumble bees than sulfoxaflor.

There are, of course, further caveats to our conclusion. Firstly, we only exposed bees through sugar water, while pollen is known to have significantly higher contamination levels^73^, and thus may lead to greater exposure in the field. Secondly, we performed behavioural observations while bumble bees had ad libitum access to the spiked solutions, contrary to Stanley et al.^42^, who observed behavioural changes after the exposure period to thiamethoxam was over. This was due to the fact that sulfoxaflor concentrations decrease quickly^57,74^, and although this may not have impacted the delivery of pollination services to field beans, having ad libitum access to gravity feeders might have discouraged workers from exiting the colonies and start foraging, particularly with adverse weather conditions. Thirdly, our bees were not immediately exposed to the peak concentration of sulfoxaflor, instead we started our exposure one day after the application. This decision was made based on the common practice of spraying blooming crops at dusk, when bees are least active, assuming that bees would not be exposed immediately after spraying also in a fully field-realistic scenario. However, once our exposure began, the peak sulfoxaflor levels started to quickly tail off, dropping from 0.047 mg/kg to below 0.004 mg/kg in just over three days. Hence, it remains possible that longer-term exposure to a lower dose could be more harmful than a shorter-term, higher-level one. While in the residue study we modelled^55^, residue levels quickly dropped below even 5 µg/kg (2.8 µg/kg five days after application and 1.8 µg/kg six days after), other residue studies have found 5 µg/kg for 14 days, so both exposure regimes represent real world scenarios (especially as sulfoxaflor can be sprayed more than once per crop). It is noteworthy that the residue studies which support either exposure regime assume a worst-case scenario where the crop is sprayed during bloom, which is not legal in the EU for sulfoxaflor products, although this is legal for some crops outside the EU. Whether such residue levels still exist with appropriate mitigation measures is unknown, however it is likely that the mitigation measure would further reduce the residue levels. This makes the exposure we modelled conservative.

The lack of interactions between *C. bombi* and sulfoxaflor in any of our metrics adds to results from previous studies^70^, which similarly found no interaction between this parasite and a range of pesticides, including clothianidin, thiamethoxam^52^, and glyphosate^20,75^. While no study has yet tested the impacts of combined *C. bombi* and pesticide exposure on a hibernating queen, when *C. bombi* is most impactful, these combined results do suggest that *C. bombi* does not meaningfully interact with many pesticides.

There is no indication of power limitation in our colony development study, with 15 to 17 colonies per treatment, and no unsupported trends in the data. However, adverse weather conditions during the semi-field experiment did limit data collection and the production of a balanced dataset, particularly for individual-level observations (see Table 1), although again there were no obvious trends. As the treatment day effect was significant for the visitation rate metric, it is reasonable to assume that the experiment was sufficiently sensitive to detect treatment effect differences, if they had been present.

Nectar-robbing behaviour of bumble bees on field beans has often been reported (e.g.^76^), and it was also noticed during our experiment. Since *Bombus terrestris* are shorter-tongued than some other bumble bee species, it is possible they are not always able to fully exploit the long-tubed *V. faba* flowers, reducing their foraging efficiency^77^ and leading to nectar-robbing, which requires lower effort and provides a higher nectar reward than that obtained by legitimate visitations^78^. However, it has been shown that *B. terrestris* are effective pollinators of field beans even after only a few flower visits^31^, and that, although nectar robbing can make field bean pollination less effective, it can still lead to higher pod production than non-pollinated flowers^59^. The absence of a ‘no-pollination’ control group in our experimental set-up means we cannot test this directly, or test whether any other yield limiting factor was preventing us detecting an effect of pollination by bees. However, our experimental plants were grown under similar conditions to those of previous experiments, where other inputs (*e.g.,* nutrients, water) were not limiting, and the plants were highly responsive to pollination treatments^31,79^.

After our experiment concluded, the European Commission implemented a ban on outdoor use of sulfoxaflor^80^ because of its risks, particularly its risk to bees^81^, finding it appropriate to allow its use only in permanent greenhouses. However, sulfoxaflor is still widely authorised globally. Our results add clarity to the understanding of how sulfoxaflor impacts bumble bee pollination and colony development, however our testing uses only one species (*B. terrestris*), with just one exposure scenario, and it should not be used to generalise across species or exposures. Exposure at different life cycle stages, or to a longer-term, lower-level cumulative exposure, may still be harmful^47^. While our study contributes to addressing the data gap, considerably more research, in particular field and semi-field work, is needed for the risk to bumble bees to be sufficiently clarified. Additionally, caution should be taken in interpreting the lack of an interaction with the parasite, *C. bombi*. This result should not be generalised across all parasites, as there is considerable diversity between parasites and parasite-pesticide interactions^19,21^.

Extrapolating from our results requires caution. Bee species have frequently been shown to differ in sensitivity to pesticides^82–85^, and consequently in experiencing sub-lethal effects^47^. For example, Boff et al.^85^ found that *Osmia bicornis* exposed to field-realistic doses of sulfoxaflor showed changes in foraging behaviour, including the number of flower visits and flight performance, suggesting that impacts of this agrochemical on pollination services supplied by different bee species may vary. Moreover, in conventional agriculture, it is common practice to use several agrochemicals to control pests and increase crop yield^86^, as well multiple sprays of the same chemical. Therefore, it cannot be excluded that sulfoxaflor might interact with other pesticides, affecting bee health or behaviour. For example, Azpiazu et al.^87^ found that sulfoxaflor decreased the survival of both *Apis mellifera* and *Osmia bicornis* when in conjunction with the fungicide fluxapyroxad, despite not having an individual impact on *B. terrestris*. Finally, there is substantial variability in how pesticides interact with different parasites (reviewed in Yordanova et al.^88^). For example, Siviter et al.^49^ observed that simultaneous exposure to sulfoxaflor and *Nosema bombi* increased *B. terrestris* mortality, while exposure to either stressor in isolation did not lead to the same effect. Future studies utilising other bee species and additional pesticides and parasites are clearly needed.

In conclusion, we found no impacts of chronic sulfoxaflor exposure, *C. bombi,* or their combination on bumble bee colony foraging behaviour, individual bee foraging behaviour, the pollination services they provide, or colony development. This is encouraging, as it may potentially indicate that the successors to neonicotinoids are less harmful to wild pollinating species like *Bombus terrestris*. However, more research is needed if we are to conclude that sulfoxaflor is safe for bees, in particular for solitary bee species.

### Supplementary Information

Supplementary Information.

## Data Availability

The datasets used and/or analysed during the current study available from the corresponding author on reasonable request.
