# Supplementary material for: Neither sulfoxaflor, Crithidia bombi, nor their combination impact bumble bee colony development or field bean pollination
Source: Sci Rep. 2023 Sep 30;13:16462. doi: 10.1038/s41598-023-43215-6 (PMC10542809; doi:10.1038/s41598-023-43215-6)
Supplement: Supplementary file 1 — Supplementary Information. [file 41598_2023_43215_MOESM1_ESM.docx]

**Neither sulfoxaflor, *Crithidia bombi*, nor their combination impact bumble bee colony development or field bean pollination**

*Edward A. Straw^1,2*^, Elena Cini^3*~^, Harriet Gold^4^, Alberto Linguadoca^2,5^, Chloe Mayne^6^, Joris Rockx^3^, Mark J.F. Brown^2^^, Michael Garratt^3^^, Simon G. Potts^3^^, Deepa Senapathi^3^~^*

* Co-First Authors

*^~^* Corresponding authors: [Elena.Cini.EC@gmail.com](mailto:Elena.Cini.EC@gmail.com) and g.d.senapathi@reading.ac.uk

^ Co-Last Authors

^1^Department of Botany, Trinity College Dublin, Dublin, Ireland, D02 PN40

^2^Centre for Ecology, Evolution and Behaviour, Department of Biological Sciences, School for Life Sciences and the Environment, Royal Holloway University of London, Egham, Surrey, TW20 0EX, UK

^3^Centre for Agri-Environmental Research, School of Agriculture, Policy and Development, University of Reading, Reading RG6 6AR, UK

^4^The School of Archaeology, Geography and Environmental Sciences, University of Reading, Reading RG6 6AB, UK

^5^Pesticides Peer Review unit, European Food Safety Authority (EFSA), Via Carlo Magno 1A, 43126 Parma, Italy

^6^School of Biological Sciences, University of Reading, Reading RG6 6AS, UK

**Supplementary methods**

**Pesticide mixing**

At Royal Holloway pure sulfoxaflor active ingredient was purchased from Greyhound Chromatograph (Merseyside, UK). The sulfoxaflor powder was diluted water and then frozen in aliquots at 200mg/L. These aliquots were defrosted, vortexed and diluted 1:10 in distilled water to a concentration of 20mg/L. This solution was then used to produce 15mL ready to dilute stock solutions at four different concentrations in 15mL Falcon tubes, labelled according to the concentration and treatment. These tubes were wrapped in tin foil to prevent photodegradation, bagged and frozen. They were shipped to the University of Reading on ice and immediately moved to the freezer. On the day of exposure, the ready to dilute stock solution was defrosted, vortexed and mixed into water 270mL and 120g sugar to produce a spiked feeding solution at the specified concentration and 30% w/w sucrose. Colonies were fed *ad libitum* spiked sucrose solution from gravity feeders.

**Plant growth conditions**

Field bean (FB) plants were used to assess the impact of treatments on bumblebee foraging behaviour, as they are economically important insect-pollinated crops for which bumblebees are effective pollinators (Garratt et al., 2014). Spring field beans of the ‘Fuego’ variety supplied by commercial seed company Limagrain UK were used for the study. Beans were sown in 3L pots containing ‘John Innes n° 2’ compost and thinned to one plant per pot when they reached an adequate size. All relevant institutional, national, and international guidelines and legislation was adhered to in the purchase and rearing of these plants. No wild plants were used, nor any endangered species.

Three hundred and twenty FB plants were grown in two temporal cohorts to ensure plants at the appropriate flowering stage were used for the experiments. Plants were grown in a glasshouse and moved to pollinator-free flight cages when in flower, and were selected to have enough fresh flowers for each day of bee visit monitoring.

**Colony development**

All metrics of colony development, bar number of pupae, are presented as the total weight of the caste/life history stage, rather than number of individuals in that caste/life history stage. This is because the weight better reflects the reproductive investment. Pupae were counted prior to eclosion, and extracting them for weighing was too difficult, hence they are presented as count data.

*Crithidia* prevalence is analysed for the individual and colony observations as well as pollination services, but not for colony development. This is because the measure of prevalence was taken two days prior to the foraging/pollination experimentation, but nearly six weeks before the colonies were frozen. So the salience of this measure will have decreased over time as secondary infections occur.

**Over-pollination**

The following observations were performed on pilot colonies foraging on field bean plants prior to the experiment to see how much time, on average, bees would require to visit the whole plant (*i.e.*, all its flowers) once. To ensure the duration of colony observations would not lead to over-pollination during the experiment, we accounted for (a) the number of flowers on a plant, and (b) the number of visits to that plant. Pilot observations were performed for 5-10 minutes during warm, sunny days.

**Supplementary table S1.** 17 April 2021: pilot observations performed for 5-10 minutes.

| **Colony 1** | **Colony 2** | **Colony 3** |
| --- | --- | --- |
| Plant 1: 37 flowers  Visits: 7  7:37=x:1  X=0.19  0.19:10=1:x  X=52.63 min to visit the whole plant once  Plant 2: 89 flowers  Visits: 5  5:89=x:1  X=0.06  0.06:10=1:x  X=166.67 min to visit the whole plant once  Mean=109.65 min | Plant 1: 41 flowers  Visits: 6  6:41=x:1  X= 0.15  0.15:10=1:x  X=66.6 min to visit the whole plant once  Plant 2: 17 flowers  Visits: 1  1:17=x:1  X=0.06  0.06:10=1:x  X=166.67 min to visit the whole plant once  Mean=116.64 min | Plant 1: 38 flowers  Visits: 3  3:38=x:1  X=0.08  0.08:10=1:x  X=125 min to visit the whole plant once  Plant 2: 62  Visits: 4  4:62=x:1  X=0.06  0.06:10=1:x  X=166.67 min to visit the whole plant once  Mean=145.84 min |

**Supplementary table S2.** 18 April 2021: pilot observations performed for 5 minutes.

| **Colony 1** | **Colony 2** |
| --- | --- |
| Plant 1: 40 flowers  Visits: 7  7:40=x:1  X=0.175  0.18:5=1:x  X= 27.78 min to visit the whole plant once  Plant 2: 40 flowers  Visits: 5  5:40=x:1  X= 0.13  0.13:5=1:x  X= 38.46 min to visit the whole plant once  Mean=33.12 min | Plant 1: 50 flowers  Visits: 6  6:50=x:1  X=0.12  0.12:5=1:x  X= 41.66 min to visit the whole plant once  Plant 2: 30 flowers  Visits: 1  1:30=x:1  X=0.03  0.03:5=1:x  X=166.67 min to visit the whole plant once  Mean=104.17 min |

**Supplementary table S3.** 21 April 2021: pilot observations performed for 5 minutes.

| **Colony 1** | **Colony 4** |
| --- | --- |
| Plant 1: 80 flowers  Visits: 18  18:80=x:1  X=0.23  0.23:5=1:x  X= 21.74 min to visit the whole plant once    Plant 2: 50 flowers  Visits: 9  9:50=x:1  X= 0.18  0.18:5=1:x  X= 27.78 min to visit the whole plant once  Mean=24.76 min | Plant 1: 114 flowers  Visits: 5  5:114=x:1  X=0.04  0.04:5=1:x  X= 125 min to visit the whole plant once    Plant 2: 86 flowers  Visits: 2  2:86=x:1  X=0.02  0.02:5=1:x  X=250 min to visit the whole plant once  Mean=187.50 min |

Considering that (a) the average number of flowers on plants was approximately 55, and (b) the average time required to visit the whole plant was approximately 100 minutes, we concluded we would have been able to safely expose plants to colonies for approximately 75 minutes over 3 days (25 minutes a day, of which 10 for acclimatisation and 15 for observations).

**Experimental set-up**

**Supplementary figure S1.** Cage arrangements for colony-level observations.

**Supplementary figure S2.** Cage arrangements for individual-level observations.

**Supplementary statistical testing**

**Individual and colony observations**

A further statistical analysis was carried out with the same response variables, but including only observations on *Crithidia*-infected colonies (*i.e.,* ‘Crithidia only’ and ‘Crithidia + sulfoxaflor’ groups, n individuals=76, n colony observations for visitation rate=45, n colony observations for number of leaves and returns=32), in order to assess any potential impact given by different percentages of *Crithidia bombi* infection on bee behaviour. The same random and fixed factors were used, except ‘treatment’ was replaced by ‘% *Crithidia* *bombi* infection’.

**Behavioural observations**

Observers were assigned to one cage each following a rotation scheme that allowed each observer to cover 2-4 cages per day, so that by the end of the day all cages would have been observed. Observers always started their observations from a different cage to further minimise any observer effect. Due to time constraints, it was often necessary for one observer to assess additional treatments to those on their personal rotation scheme, and weather conditions did not always allow all colonies to be observed every day.

**Colony observations**

Plants were placed in a randomised order and observations started from the right to the left. When observations had ended, all bees were returned to colonies ready for the next day of observations and plants were moved out of the cages into a pollinator-free cage to prevent over-pollination. This allowed all plants in different cages to be exposed to colonies for the same amount of time.

**Individual observations**

If the bee did not start foraging within 10 minutes, it was captured and another bee was allowed out of the colony. If the bee attempted to return to the colony before 15 minutes had elapsed (*i.e.,* landing on the entrance), it was assumed the foraging trip had ended. At the end of the trip, the bee was captured and returned to the colony only after all individual observations had been completed to prevent pseudo-replications.

**Pollination services**

Once again, a further statistical analysis was performed with plants exposed to *Crithidia*-infected colonies replacing ‘treatment’ with ‘% *Crithidia* *bombi* infection’ as fixed term. In this case, as we aimed to investigate yield data and not changes in bee behaviours, plants exposed to control colonies were also included in the analysis (n=97).

**Supplementary Results**

|  | **Worker weight** | | **Larval weight** | | **Drone weight** | | **Number of pupae** | |
| --- | --- | --- | --- | --- | --- | --- | --- | --- |
| **Treatment** | ***n=*** | **grams ± SD** | ***n=*** | **grams ± SD** | ***n=*** | **grams ± SD** | ***n=*** | **Count ± SD** |
| **Control** | 16 | 15.000 ± 6.889g | 16 | 4.791 ± 3.373g | 15 | 1.526 ± 1.729g | 16 | 24.7 ± 15.3 |
| **Sulfoxaflor** | 15 | 13.773 ± 6.368g | 15 | 7.679 ± 5.079g | 14 | 0.630 ± 0.644g | 15 | 25.1 ± 9.6 |
| ***C. bombi*** | 17 | 14.060 ± 5.889g | 16 | 6.548 ± 4.295g | 17 | 1.457 ± 1.366g | 17 | 25.1 ± 9.6 |
| **Sulfoxaflor and *C. bombi*** | 17 | 14.689 ± 6.228g | 17 | 5.209g ± 2.989g | 17 | 1.335 ± 1.375g | 17 | 21.4 ± 17.2 |

**Supplementary table S4.** Colony Development.

**Supplementary table S5.** Post Hoc testing on Larval Weight. P-values are presented and Benjamini-Hochberg corrected.

|  | Control | Sulfoxaflor + Crithidia | Sulfoxaflor |
| --- | --- | --- | --- |
| Crithidia | 0.67 | 0.73 | 0.73 |
| Sulfoxaflor | 0.67 | 0.67 | - |
| Sulfoxaflor + Crithidia | 0.73 | - | - |

**Supplementary table S6.** Post Hoc testing on Drone Weight. P values are presented and Benjamini-Hochberg corrected.

|  | Control | Sulfoxaflor + Crithidia | Sulfoxaflor |
| --- | --- | --- | --- |
| Crithidia | 0.94 | 0.94 | 0.71 |
| Sulfoxaflor | 0.92 | 0.71 | - |
| Sulfoxaflor + Crithidia | 0.94 | - | - |

**Colony behaviour**

No effect prevalence of *Crithidia* infection was found on visitation rate or the number of bees leaving and returning to colonies

**Individual behaviour**

Statistical analysis of *Crithidia*-infected colonies did not show any significant effect of the percentage of *Crithidia* infection on foraging rate (F_1,68.9_=0.70, p=0.405), pollen collection (F_1,13.3_=0.82, p=0.382), duration of foraging trip (F_1,12.7_=0.42, p=0.528), latency (F_1,69.9_=0.01, p=0.918), average time between visits (F_1,15.2_=1.29, p=0.273), and average duration of flower visit (F_1,69.4_=1.05, p=0.308), and no effect of observation day or interaction with treatment was found on any response variable.

**Pollination services**

Final models of plant yield measurements did not show any significant effect of treatment or percentage of *Crithidia bombi* infection on average number of beans (F_1,24.8_=0.18, p=0.673), average number of pods (F_1,28.6_=1.99, p=0.170), average pod weight (F_1,65_=0.00, p=0.970) and average bean weight (F_1,21.7_=0.23, p=0.637). No effect of location of first node on average number of beans (all treatments: F_2,68.3_=0.10, p=0.907; *Crithidia* and control only: F_2,63.9_=0.50, p=0.610), pods (all treatments: F_2,92.4_=0.19, p=0.831; *Crithidia* and control only: F_2,85.2_=1.04, p=0.357), pod weight (all treatments: F_2,67.7_=0.51, p=0.604, *Crithidia* and control only: F_2,65_=0.02, p=0.981) and bean weight (all treatments: F_2,63.5_=0.88, p=0.421; *Crithidia* and control: F_2,63.5_=0.28, p=0.760) was found.

| **Supplementary table S7.** Results of final models with lowest AIC within ΔAIC of 2. Significant p-values (≤0.050) are highlighted in bold. | | | | | | |
| --- | --- | --- | --- | --- | --- | --- |
| **Colony-level observations: Analysis of all treatment colonies** | | | | | | |
| **Response variable** | **Fixed effect in selected model** | **Estimates** | **SE** | **F_ndf,ddf_ value** | ***p*-value** | ***R^2^*** |
| **Visitation rate** | Observation day 2 | 0.02627 | 0.00745 | F_2,53.9_=9.43 | **<0.001** | **18.34** |
|  | Observation day 3 | 0.02837 | 0.00713 |  |  |  |
| **N bees leaving colony** | Observation day 2 | 1.42300 | 1.11700 | χ^2^_2_=8.33 | **0.016** | **4.23** |
|  | Observation day 3 | 1.72000 | 1.05500 |  |  |  |
| **N bees returning to colony** | Observation day 2 | 1.27100 | 1.11900 | χ^2^_2_=1.36 | 0.507 | 12.82 |
|  | Observation day 3 | 1.78400 | 1.05200 |  |  |  |
|  | Crithidia | 1.66300 | 1.15200 | χ^2^_3_=1.51 | 0.680 |  |
|  | Sulfoxaflor | 1.57700 | 1.16200 |  |  |  |
|  | Crithidia*sulfoxaflor | 1.45000 | 1.17000 |  |  |  |
|  | Observation day 2*Crithidia | -1.28350 | 1.28970 | χ^2^_6_=2.28 | 0.892 |  |
|  | Observation day 2*Sulfoxaflor | -1.06230 | 1.29070 |  |  |  |
|  | Observation day 2*Crithidia*Sulfoxaflor | -1.44530 | 1.33830 |  |  |  |
|  | Observation day 3*Crithidia | -1.30950 | 1.20390 |  |  |  |
|  | Observation day 3*Sulfoxaflor | -1.57570 | 1.22470 |  |  |  |
|  | Observation day 3*Crithidia*Sulfoxaflor | -1.47340 | 1.23030 |  |  |  |

| **Supplementary table S8.** Results of final models with lowest AIC within ΔAIC of 2. Significant p-values (≤0.050) are highlighted in bold. | | | | | | |
| --- | --- | --- | --- | --- | --- | --- |
| **Colony-level observations: Analysis of Crithidia-infected colonies** | | | | | | |
| **Response variable** | **Fixed effects in selected model** | **Estimates** | **SE** | **F_ndf,ddf_ value** | ***p*-value** | ***R^2^*** |
| **Visitation rate** | Observation day 2  Observation day 3 | 0.03147  0.03371 | 0.00948  0.00897 | F_2,25.5_=8.39 | **0.002** | **29.56** |
| **N bees leaving colony** | Observation day 2  Observation day 3 | -0.11499  0.16684 | 0.21519  0.19955 | χ^2^_2_=2.93 | 0.231 |  |
|  | % Crithidia | 0.00763 | 0.02418 | χ^2^_1_=0.30 | 0.587 | 10.81 |
|  | Observation day 2*% Crithidia  Observation day 3*% Crithidia | 0.00155  -0.00005 | 0.03019  0.02402 | χ^2^_2_=0.00 | 0.998 |  |
| **N bees returning to colony** | Observation day 2  Observation day 3 | 0.23300  0.20790 | 0.48760  0.45770 | χ^2^_2_=0.19 | 0.908 | 26.56 |
|  | % Crithidia | 0.07085 | 0.06915 | χ^2^_1_=0.56 | 0.455 |  |
|  | Observation day 2*% Crithidia  Observation day 3*% Crithidia | -0.21430  -0.09420 | 0.09390  0.06350 | χ^2^_2_=5.20 | 0.074 |  |

| **Supplementary table S9.** Results of final models with lowest AIC within ΔAIC of 2. | | | | | | | | | | | |
| --- | --- | --- | --- | --- | --- | --- | --- | --- | --- | --- | --- |
| **Individual-level observations: Analysis of all treatment colonies** | | | | | | | | | | | |
| **Response variable** | **Fixed effects** | **Estimates** | **SE** | **F_ndf,ddf_** | | ***p*-value** | | | ***R^2^*** | |  |
| **Foraging rate** | Observation day 2  Observation day 3 | 1.18720  -0.04270 | 0.74500  0.72710 | F_2,129.6_=0.49 | | 0.614 | | | 8.69 | |  |
|  | Crithidia  Sulfoxaflor  Crithidia*sulfoxaflor | 0.03570  0.51640  -0.89190 | 0.84300  0.88490  1.88870 | F_3,21.5_=0.51 | | 0.677 | | |  |  |  |
|  | Observation day 2*Crithidia  Observation day 2*Sulfoxaflor  Observation day 2*Crithidia*Sulfoxaflor  Observation day 3*Crithidia  Observation day 3*Sulfoxaflor  Observation day 3*Crithidia*Sulfoxaflor | -1.72470  -1.63780  0.29010  1.23180  -0.42300  0.09560 | 1.09950  1.17400  1.12490  1.06220  1.17930  1.15920 | F_6,102.7_=1.74 | | 0.119 | | |  |  |  |
| **Foraging trip duration** | Observation day 2  Observation day 3 | -1.10040  0.58790 | 1.31000  1.25610 | F_2,124.2_=0.56 | | 0.575 | | | 8.99 | |  |
|  | Crithidia  Sulfoxaflor  Crithidia*sulfoxaflor | 0.01600  -3.40100  -1.61100 | 1.62100  1.69000  1.70600 | F_3,20.2_=0.81 | | 0.504 | | |  |  |  |
|  | Observation day 2*Crithidia  Observation day 2*Sulfoxaflor  Observation day 2*Crithidia*Sulfoxaflor  Observation day 3*Crithidia  Observation day 3*Sulfoxaflor  Observation day 3*Crithidia*Sulfoxaflor | 1.40400  2.94000  3.27600  -2.47400  2.31700  0.38300 | 1.94700  2.08300  1.97700  1.84600  2.07900  2.01700 | F_6,109.9_=1.66 | | 0.138 | | |  |  |  |
| **Latency** | Observation day 2  Observation day 3 | 0.02210  -0.43150 | 0.90450  0.89600 | F_2,135_=0.61 | | 0.545 | | | 5.74 | |  |
|  | Crithidia  Sulfoxaflor  Crithidia*sulfoxaflor | -0.02770  -0.21540  0.92050 | 0.96670  1.02750  1.01750 | F_3,136.3_=1.11 | | 0.346 | | |  |  |  |
|  | Observation day 2*Crithidia  Observation day 2*Sulfoxaflor  Observation day 2*Crithidia*Sulfoxaflor  Observation day 3*Crithidia  Observation day 3*Sulfoxaflor  Observation day 3*Crithidia*Sulfoxaflor | 0.77190  1.31330  -0.29710  1.58810  0.16000  -0.01790 | 1.34380  1.43540  1.37070  1.30240  1.43070  1.42140 | F_6,111.9_=0.63 | | 0.707 | | |  |  |  |
| **Time between visits** | Observation day 2  Observation day 3 | -0.02399  -0.10942 | 0.17626  0.17464 | F_2,135.1_=0.18 | | 0.836 | | | 3.34 | |  |
|  | Crithidia  Sulfoxaflor  Crithidia*sulfoxaflor | -0.17545  -0.04468  0.17248 | -0.18839  0.20012  0.19831 | F_3,136.3_=0.74 | | 0.527 | | |  |  |  |
|  | Observation day 2*Crithidia  Observation day 2*Sulfoxaflor  Observation day 2*Crithidia*Sulfoxaflor  Observation day 3*Crithidia  Observation day 3*Sulfoxaflor  Observation day 3*Crithidia*Sulfoxaflor | 0.09269  0.02529  -0.22952  0.13965  0.20741  -0.07824 | 0.26172  0.27952  0.26710  0.25384  0.27881  0.27702 | F_6,111.8_=0.35 | | 0.906 | | |  |  |  |
| **Duration of flower visits** | Observation day 2  Observation day 3 | -0.02407  0.02859 | 0.03900  0.03843 | F_2,131_=0.53 | | 0.588 | | | 5.36 | |  |
|  | Crithidia  Sulfoxaflor  Crithidia*sulfoxaflor | 0.02359  -0.04060  0.03144 | 0.04279  0.04486  0.04514 | F_3,20.6_=0.68 | | 0.576 | | |  |  |  |
|  | Observation day 2*Crithidia  Observation day 2*Sulfoxaflor  Observation day 2*Crithidia*Sulfoxaflor  Observation day 3*Crithidia  Observation day 3*Sulfoxaflor  Observation day 3*Crithidia*Sulfoxaflor | 0.03384  0.07551  -0.04483  -0.01327  0.03866  0.00541 | 0.05720  0.06093  0.05892  0.05597  0.06167  0.06102 | F_6,90.3_=0.77 | | 0.596 | | |  |  |  |
| **Pollen collection** | Observation day 2  Observation day 3 | -1.41750  -0.69850 | 0.96490  0.96600 | F_2,124.9_=0.73 | | 0.484 | | | 19.95 | |  |
|  | Crithidia  Sulfoxaflor  Crithidia*sulfoxaflor | -0.04800  -0.94700  -3.41900 | 1.32200  1.31300  1.32700 | F_3,19.7_=0.89 | | 0.463 | | |  |  |  |
|  | Observation day 2*Crithidia  Observation day 2*Sulfoxaflor  Observation day 2*Crithidia*Sulfoxaflor  Observation day 3*Crithidia  Observation day 3*Sulfoxaflor  Observation day 3*Crithidia*Sulfoxaflor | -1.15130  0.92330  3.88850  -1.14740  0.76910  1.41350 | 1.39810  1.45910  1.41390  1.39740  1.54740  1.44310 | F_6,35.6_=2.35 | | 0.051 | | |  |  |  |
| **Supplementary table S10.** Results of final models with lowest AIC within ΔAIC of 2. | | | | | | | |  | |  |  |
| **Individual-level observations: Analysis of *Crithidia*-infected colonies** | | | | | | | | | |  |  |
| **Response variable** | **Fixed effects** | **Estimates** | **SE** | **F_ndf,ddf_** | ***p*-value** | | ***R2*** | |  |  |  |
| **Foraging rate** | Observation day 2  Observation day 3 | 0.18990  0.53470 | 0.65330  0.67980 | F_2,69.1_=0.38 | 0.685 | | 2.13 | |  |  |  |
|  | % Crithidia | 0.04518 | 0.07267 | F_1,68.9_=0.70 | 0.405 | |  |  |  |  |  |
|  | Observation day 2*% Crithidia  Observation day 3*% Crithidia | -0.02167  -0.00848 | 0.09317  0.11184 | F_2,69.7_=0.03 | 0.972 | |  |  |  |  |  |
| **Foraging trip duration** | Observation day 2  Observation day 3 | 0.96040  -0.75820 | 1.01900  1.07230 | F_2,64.7_=1.65 | 0.199 | | 5.33 | |  |  |  |
|  | % Crithidia | -0.04512 | 0.12400 | F_1,12.7_=0.42 | 0.528 | |  |  |  |  |  |
|  | Observation day 2*% Crithidia  Observation day 3*% Crithidia | -0.02907  0.04402 | 0.14670  0.17802 | F_2,67_=0.11 | 0.898 | |  |  |  |  |  |
| **Latency** | Observation day 2  Observation day 3 | 0.35440  0.50540 | 0.70350  0.73380 | F_2,69.7_=0.30 | 0.744 | | 2.14 | |  |  |  |
|  | % Crithidia | 0.06228 | 0.07853 | F_1,69.9_=0.01 | 0.918 | |  |  |  |  |  |
|  | Observation day 2*% Crithidia  Observation day 3*% Crithidia | -0.09604  -0.05287 | 0.09995  0.12025 | F_2,69.6_=0.46 | 0.631 | |  |  |  |  |  |
| **Time between visits** | Observation day 2  Observation day 3 | -0.07851  -0.07013 | 0.08820  0.09221 | F_2,66.6_=0.61 | 0.546 | | 5.56 | |  |  |  |
|  | % Crithidia | -0.01679 | 0.01013 | F_1,15.2_=1.29 | 0.273 | |  |  |  |  |  |
|  | Observation day 2*% Crithidia  Observation day 3*% Crithidia | 0.01320  0.01763 | 0.01261  0.01520 | F_2,68.4_=0.80 | 0.452 | |  |  |  |  |  |
| **Duration of visits** | Observation day 2  Observation day 3 | -0.01492  -0.02310 | 0.02999  0.03123 | F_2,69.4_=0.35 | 0.704 | | 2.61 | |  |  |  |
|  | % Crithidia | -0.00274 | 0.00334 | F_1,69.4_=1.05 | 0.308 | |  |  |  |  |  |
|  | Observation day 2*% Crithidia  Observation day 3*% Crithidia | 0.00104  0.00170 | 0.00427  0.00513 | F_2,69.9_=0.06 | 0.943 | |  |  |  |  |  |
| **Pollen collection** | Observation day 2  Observation day 3 | -0.35140  -0.96520 | 0.71090  0.74560 | F_2,64.9_=0.71 | 0.494 | | 8.25 | |  |  |  |
|  | % Crithidia | 0.12460 | 0.09923 | F_1,13.3_=0.82 | 0.382 | |  |  |  |  |  |
|  | Observation day 2*% Crithidia  Observation day 3*% Crithidia | -0.07581  -0.13262 | 0.10911  0.12472 | F_2,68_=0.57 | 0.571 | |  |  |  |  |  |

| **Supplementary table S11.** Results of final models with lowest AIC within ΔAIC of 2. | | | | | | |  | |
| --- | --- | --- | --- | --- | --- | --- | --- | --- |
| **Plant yield measurements: Analysis of plants exposed to all treatment colonies** | | | | | | | | |
| **Response variable** | **Fixed effects** | **Estimates** | **SE** | **F_ndf,ddf_** | ***p*-value** | ***R^2^*** | |  |
| **Average number of beans** | Crithidia  Sulfoxaflor  Crithidia*sulfoxaflor | -0.39440  -0.04630  0.34400 | 0.29960  0.30600  0.30530 | F_3,27_=1.94 | 0.147 | 8.01 | |  |
|  | Middle nodes  Late nodes | 0.08130  -0.04964 | 0.22833  0.36775 | F_2,68.3_=0.10 | 0.907 |  |  |  |
| **Average number of pods** | Crithidia  Sulfoxaflor  Crithidia*sulfoxaflor | -1.01140  -0.78430  -0.59360 | 0.47800  0.47680  0.47810 | F_3,31.6_=1.63 | 0.203 | 4.99 | |  |
|  | Middle nodes  Late nodes | 0.02110  0.28560 | 0.26650  0.47920 | F_2,92.4_=0.19 | 0.831 |  |  |  |
| **Average pod weight** | Crithidia  Sulfoxaflor  Crithidia*sulfoxaflor | -0.18905  0.06726  0.04162 | 0.24375  0.24802  0.24720 | F_3,24.9_=0.33 | 0.803 | 2.83 | |  |
|  | Middle nodes  Late nodes | -0.13510  -0.24090 | 0.17420  0.27890 | F_2,67.7_=0.51 | 0.604 |  |  |  |
| **Average bean weight** | Crithidia  Sulfoxaflor  Crithidia*sulfoxaflor | -0.06640  0.01029  -0.05345 | 0.07744  0.07832  0.07780 | F_3,24.7_=0.43 | 0.734 | 4.22 | |  |
|  | Middle nodes  Late nodes | -0.03161  -0.09923 | 0.04796  0.07595 | F_2,63.5_=0.88 | 0.421 |  |  |  |

| **Supplementary table S12.** Results of final models with lowest AIC within ΔAIC of 2. | | | | | | |  | |
| --- | --- | --- | --- | --- | --- | --- | --- | --- |
| **Plant yield measurements: Analysis of plants exposed to Crithidia-infected and control colonies** | | | | | | | | |
| **Response variable** | **Fixed effects** | **Estimates** | **SE** | **F_ndf,ddf_** | ***p*-value** | ***R^2^*** | |  |
| **Average number of beans** | % Crithidia | 0.00236 | 0.00591 | F_1,24.8_=0.18 | 0.673 | 1.78 | |  |
|  | Middle nodes  Late nodes | 0.15440  0.39550 | 0.25350  0.41460 | F_2,63.9_=0.50 | 0.610 |  |  |  |
| **Average number of pods** | % Crithidia | -0.01460 | 0.01025 | F_1,28.6_=1.99 | 0.170 | 4.47 | |  |
|  | Middle nodes  Late nodes | 0.60840  -0.07170 | 0.43680  0.79910 | F_2,85.2_=1.04 | 0.357 |  |  |  |
| **Average pod weight** | % Crithidia | 0.00107 | 0.00372 | F_1,65_=0.00 | 0.970 | 0.06 | |  |
|  | Middle nodes  Late nodes | 0.01917  0.05850 | 0.18685  0.30904 | F_2,65_=0.02 | 0.981 |  |  |  |
| **Average bean weight** | % Crithidia | -0.00063 | 0.00133 | F_1,21.7_=0.23 | 0.637 | 1.19 | |  |
|  | Middle nodes  Late nodes | 0.02082  -0.04053 | 0.05463  0.08845 | F_2,63.5_=0.28 | 0.760 |  |  |  |
